# AMPK: Regulating Energy Balance at the Cellular and Whole Body Levels

**DOI:** 10.1152/physiol.00050.2013

**Published:** 2014-03

**Authors:** D. Grahame Hardie, Michael L. J. Ashford

**Affiliations:** Division of Cell Signalling & Immunology, College of Life Sciences, and Division of Cardiovascular and Diabetes Medicine, College of Medicine, Dentistry & Nursing, University of Dundee, Dundee, Scotland, United Kingdom

## Abstract

AMP-activated protein kinase appears to have evolved in single-celled eukaryotes as an adenine nucleotide sensor that maintains energy homeostasis at the cellular level. However, during evolution of more complex multicellular organisms, the system has adapted to interact with hormones so that it also plays a key role in balancing energy intake and expenditure at the whole body level.

Although discovered via its ability to phosphorylate and inactivate two enzymes involved in lipid synthesis, i.e., acetyl-CoA carboxylase and HMG-CoA reductase (6, 9, 10), the AMP-activated protein kinase (AMPK) is now recognized to have dozens of downstream targets (23–25, 53) and may turn out to have hundreds. Its principal role is as an energy sensor monitoring the cellular ratios of AMP to ATP and/or ADP to ATP (20). Once switched on by stresses that increase these ratios, it activates catabolic pathways that generate ATP while switching off nonessential ATP-requiring processes, thus acting to restore energy homeostasis. Although it appears to have evolved in single-celled eukaryotes as a cell-autonomous energy sensor, in multicellular organisms, its role has adapted so that it also responds to hormones regulating whole-body energy balance. The aim of this review is to discuss these two distinct levels at which the AMPK system operates. Given the very large number of papers now published on AMPK, it is unfortunately not possible to provide comprehensive coverage.

## AMPK- and SNF1-Related Kinases: Roles in Lower Eukaryotes

**FIGURE 1.**
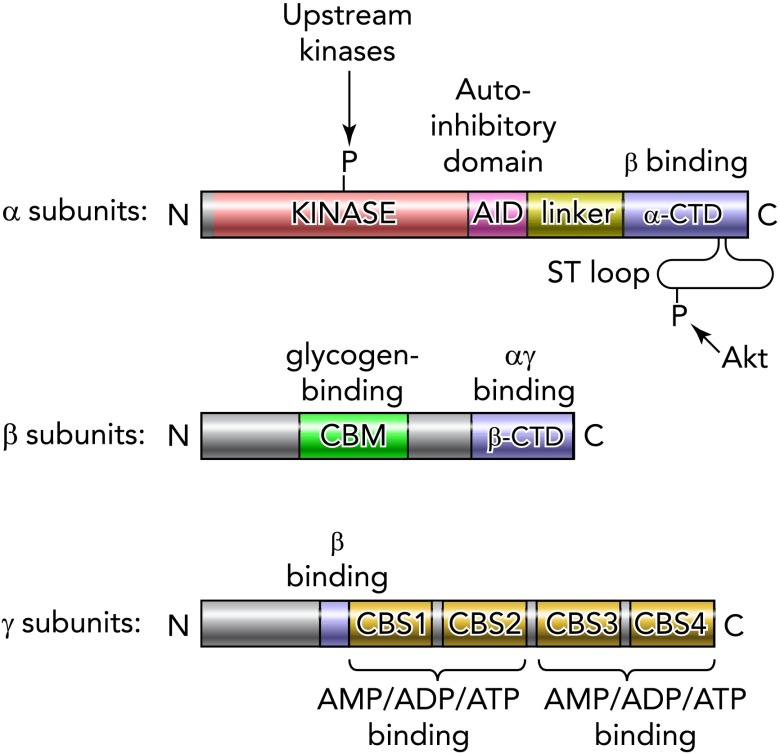
Domain structure of eukaryotic AMPK orthologs The catalytic α-subunits contain conventional serine/threonine kinase domains containing the threonine residue (Thr172 in rat α2) phosphorylated by upstream kinases. The kinase domains are followed (at least in vertebrates) by small domains with a negative effect on kinase activity (auto-inhibitory domains), which are joined to the COOH-terminal domains (α-CTD) by a less well conserved linker. In vertebrates, there is also a flexible serine-/threonine-rich loop (ST loop) within the α-CTD that is phosphorylated by Akt. The β-subunits contain two conserved regions, a carbohydrate-binding module (CBM) that causes the mammalian complex to bind to glycogen particles, and a COOH-terminal domain (β-CTD) that provides the bridge between the α- and γ-subunits. The γ-subunits contain variable NH_2_-terminal regions followed by a short sequence involved in binding to the β-subunit, then four tandem repeats of a cystathionine-β-synthase (CBS) motif. These act in pairs to form the binding sites for adenine nucleotides; in mammalian AMPK, there is one site between CBS1 and CBS2 and two between CBS3 and CBS4.

AMPK appears to exist universally as heterotrimeric complexes comprised of catalytic α-subunits and regulatory β- and γ-subunits (FIGURE 1), and well conserved genes encoding these can be found in the genomes of essentially all eukaryotes, including protists, fungi, plants, and animals (26). It is therefore likely that AMPK arose at a very early stage during eukaryotic evolution. A critical event in the genesis of eukaryotes is thought to have been the engulfment by archaeal cells of oxidative bacteria that became the mitochondria that allowed eukaryotes to generate ATP by oxidative metabolism utilizing molecular oxygen. What would have been needed at that time would have been a system to monitor the output of the primitive mitochondria and modulate their cellular content and function according to the energy demands of the host cell; this remains one of the key functions of AMPK today.

Genes encoding the α- and γ-subunits of the AMPK ortholog in the yeast *Saccharomyces cerevisiae* (*SNF1* and *SNF4*) were cloned in the 1980s (11, 12), although their relationship with AMPK was not recognized until 1994 (51, 76). If yeast are grown in batch culture in high glucose, they initially grow rapidly using only fermentation (glycolytic metabolism of glucose to ethanol) to generate ATP. However, when glucose runs low, growth pauses as genes required for mitochondrial oxidative metabolism (previously repressed by glucose) are switched on. Growth then resumes at a lower rate, a phenomenon termed the diauxic shift. The switch to oxidative metabolism allows more efficient ATP generation from the remaining glucose as well as from ethanol generated during the fermentation phase, but yeast with *snf1* or *snf4* mutations are unable to perform this switch and do not display the diauxic shift (27). They are also unable to switch on genes required for growth on other fermentable carbon sources, such as sucrose or raffinose (11, 12).

AMPK orthologs are also found in plants, where they are termed SNF1-related kinases (SnRK1 subfamily) (58). In the moss *Physcomitrella patens*, plants are viable if both SnRK1 catalytic subunits are deleted, but only if continuously illuminated; they fail to grow on a normal daily light/dark cycle (68). In the higher plant *Arabidopsis thaliana*, silencing of both SnRK1 catalytic subunits leads to a severe growth defect and failure to upregulate expression of genes required during darkness (5). Thus SnRK1 kinases are required for the response to darkness, the equivalent of starvation in plants. In the nematode worm *Caenorhabditis elegans*, the AMPK ortholog is required for the lifespan extension that occurs in response to dietary restriction and other stresses (4, 21). Taken together, these findings suggest that the ancestral role of AMPK was in the response to starvation for a carbon source.

## AMPK and SNF1 Complexes: Regulation by Adenine Nucleotides

As with many other protein kinases, AMPK and its orthologs are only significantly active when phosphorylated at a conserved threonine within the kinase domain by upstream kinases (FIGURE 1); this threonine is usually referred to as Thr172 due to its position in the rat α2-subunit sequence (29). In mammals, the principal upstream kinase phosphorylating Thr172 is a heterotrimeric complex containing the tumor suppressor kinase LKB1 (28, 64, 75, 82). This complex has a high basal activity and appears to be constitutively active (61), but its ability to phosphorylate Thr172 is enhanced by conformational changes in its substrate, AMPK, caused by binding of AMP to the γ-subunit (FIGURE 1). AMP binding also inhibits Thr172 dephosphorylation (20), and it was recently reported that this effect of AMP was mimicked by binding of ADP (78). Although this has been confirmed, the effects of ADP are 10-fold less potent than those of AMP, and the much larger increases in AMP that occur in cells treated with mitochondrial inhibitors suggest that AMP might remain the more physiologically relevant signal (20). Recent reports that ADP binding also promoted Thr172 phosphorylation (55) could not be confirmed (20).

**FIGURE 2.**
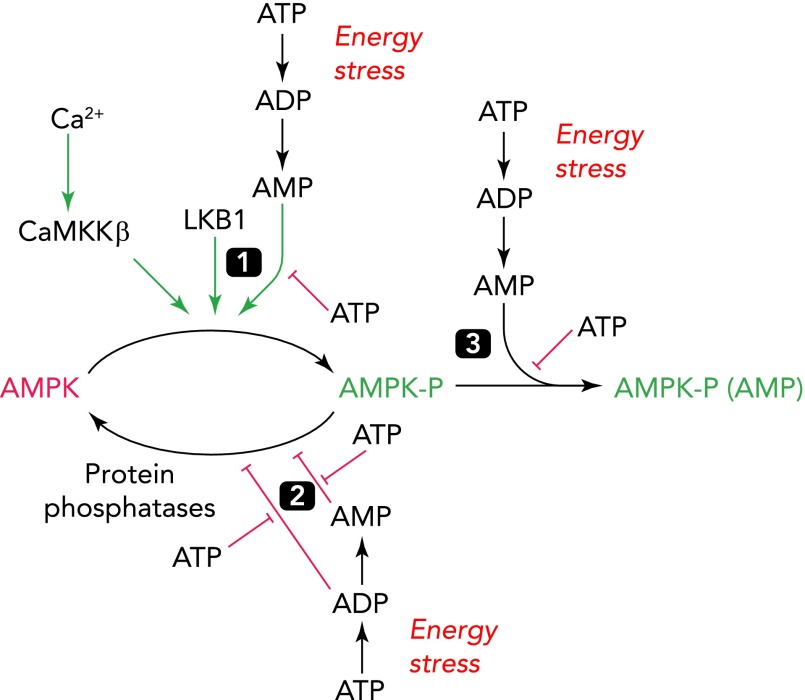
Regulation of mammalian AMPK by adenine nucleotides and Ca^2+^ Cellular energy stress leads to net conversion of ATP to ADP, some of which is converted to AMP by the adenylate kinase reaction (2ADP ↔ ATP + AMP), which maintains AMP at low levels in unstressed cells. AMP then activates AMPK by three mechanisms, all of which are due to binding to one or more sites on the AMPK-γ subunit: *1*) promoting Thr172 phosphorylation by LKB1; *2*) inhibiting Thr172 dephosphorylation by protein phosphatases; *3*) allosteric activation. Only *mechanism 2* is mimicked by ADP, and then only at higher concentrations than AMP (20). AMPK can also be activated by increases in cytosolic Ca^2+^, which triggers direct phosphorylation of Thr172 by CaMKKβ.

Via these two effects, AMP binding causes a sensitive switch to the active, phosphorylated form of AMPK. This activation is further amplified by allosteric activation, which is triggered only by AMP (FIGURE 2). The physiological significance of allosteric activation has been questioned (8, 54) on the grounds that AMP might have difficulty competing with the much higher cellular concentrations of ATP and ADP. However, recent studies show that AMP concentrations from 50 to 500 μM cause a large allosteric activation (>10-fold) in cell-free assays, even in the presence of much higher ATP concentrations (5 mM) that are within the physiological range. Cellular AMP concentrations also increased over a similar range (40–270 μM) when cultured human cells were treated with the mitochondrial inhibitor berberine (20). Furthermore, when AMPK carrying a phosphomimetic T172D mutation, which retains allosteric activation by AMP (65), was expressed in AMPK-null mouse embryo fibroblasts, the phosphorylation of the downstream target ACC (which was absent in the untransfected cells) was markedly stimulated by berberine (20). Since the T172D mutant cannot be phosphorylated at Thr172, this effect can only have been due to allosteric activation.

Thus mammalian AMPK is activated by AMP by a tripartite mechanism: *1*) promotion of Thr172 phosphorylation; *2*) inhibition of Thr172 dephosphorylation; *3*) allosteric activation. Only the second effect is mimicked by ADP, although all three are antagonized by ATP (FIGURE 2). When cells were treated with berberine, the decreases in ATP (<20%) and increases in ADP (approximately twofold) were much smaller than the increases in AMP (six- to sevenfold) (20). Thus mammalian AMPK may have evolved to respond primarily to AMP because it is the most sensitive indicator of energy stress, despite its low cellular concentration.

Surprisingly, allosteric activation by AMP is only observed in AMPK orthologs from animal species, including insects (56) and nematodes (4), and not in those from fungi (73, 76) or plants (45). The fungal and plant orthologs are still modulated by reversible phosphorylation of the residue equivalent to Thr172; in *S. cerevisiae*, phosphorylation of this residue (Thr210) increases in response to glucose starvation and correlates with large increases in cellular ADP-to-ATP and AMP-to-ATP ratios (73). Recently, it has been reported that binding of ADP, but not AMP, to the yeast SNF1 complex protects against dephosphorylation of Thr210 (48), suggesting that ADP, rather than AMP, may be the critical activating signal in fungi.

## AMPK: Regulation by Ca^2+^- and Calmodulin-Dependent Protein Kinases

Treatment of cells with agents that increase intracellular Ca^2+^ cause increased Thr172 phosphorylation, even in tumor cells lacking LKB1. This led to the realization that AMPK activation by the calmodulin-dependent protein kinase kinases (CaMKKs), first demonstrated 10 years earlier (31), was physiologically relevant, particularly with the CaMKKβ (CaMKK2) isoform (30, 34, 74) (FIGURE 2). Increases in cytosolic Ca^2+^ often trigger energy-requiring processes such as contraction or secretion and also require ATP expenditure to pump Ca^2+^ back out of the cytosol. AMPK activation by the Ca^2+^-CaMKK pathway may in part be a mechanism to anticipate this demand for ATP before it has occurred. This pathway also provides a mechanism for hormones that increase cytosolic Ca^2+^ to activate AMPK. Physiological situations where this pathway operates include depolarization of neurons (30), activation of T cells via their antigen receptor (67), and treatment of cells with agents that activate G-protein-coupled receptors (GPCRs) coupled to release of cytosolic Ca^2+^ (including ghrelin; see below).

## AMPK: Regulation by TGF-β-Activated Kinase-1

In 2006, Carlson and coworkers reported results of screening a mouse embryo library for DNAs that would restore growth on raffinose to an *S. cerevisiae* strain that lacked all three kinases upstream of the yeast AMPK ortholog (52). As well as LKB1 and CaMKKβ, this screen identified TGF-β-activated kinase-1 (TAK1) as a potential upstream kinase for AMPK. This was a surprise, because TAK1 is best known for its involvement in signaling from receptors for pro-inflammatory cytokines and from toll-like receptors involved in innate immunity (16). In the same year, Schneider's group reported that knocking out TAK1 in mouse embryo fibroblasts reduced phosphorylation of Thr172 on AMPK in response to several AMPK activators such as oligomycin or metformin (79). However, in contrast to Carlson's group, who showed that TAK1 directly phosphorylated Thr172 in cell-free assays (52), they proposed a pathway in which oligomycin or metformin activated TAK1, which in turn activated LKB1 by some unspecified mechanism, leading to Thr172 phosphorylation by LKB1. This is difficult to reconcile with the well established mechanisms described in an earlier section, where these same agents increase AMP that binds to the AMPK-γ subunit, promoting net phosphorylation by LKB1. Since 2006, there have been few further reports that help to clarify the potential link between TAK1 and AMPK, although it was reported that tumor necrosis factor-related apoptosis-inducing ligand (TRAIL) activated AMPK in cultured breast cancer cells via a mechanism that was LKB1 and CaMKKβ independent but TAK1 dependent (32).

## Regulation of AMPK by the Insulin-Activated Kinase Akt

AMPK is activated during times of energy stress, when it inhibits growth and promotes catabolism and breakdown of energy stores. By contrast, insulin is an anabolic hormone released during times of nutrient excess, which promotes growth and energy storage. It is therefore not surprising that there should be antagonism between these two pathways. The insulin-activated kinase Akt has been shown to phosphorylate the α1 catalytic subunit of AMPK at Ser485 (rat numbering). This appeared to reduce the rate of subsequent Thr172 phosphorylation and activation by LKB1 in a cell-free assay, whereas prior insulin treatment also reduced Thr172 phosphorylation during ischemia in perfused rat hearts (33). This mechanism also occurs in human hepatocytes infected with hepatitis C virus (when Akt is activated) and appears to be necessary for efficient viral replication (46). Interestingly, Ser485 lies in a loop of ∼55 residues that appears to be flexible in its unphosphorylated state, being poorly resolved in a partial crystal structure of AMPK expressed in bacteria (77). We now term this the “ST loop” (FIGURE 1) because it is highly serine/threonine rich; it is absent in orthologs from invertebrates, fungi, and plants but well conserved in vertebrates. The ST loop is also phosphorylated by PKA at Ser485 (35) and by GSK3 at other sites (66), suggesting that it may have additional regulatory functions. The AMPK-α2 isoform contains a residue equivalent to Ser485 (Ser491). Although it has not yet been definitively shown that phosphorylation of Ser491 has the same inhibitory effect on LKB1 activation, its possible role in regulating AMPK in the hypothalamus is discussed below.

## Downstream Targets of AMPK

A full discussion of downstream targets for AMPK are beyond the scope of this article, and readers are referred to other reviews (23–25, 53); we focus here mainly on some recent findings. In general, AMPK promotes catabolic pathways that generate ATP, while switching off ATP-consuming processes not essential for short-term cell survival. With respect to catabolism, AMPK acutely activates glucose and fatty acid uptake, glycolysis, and fatty acid oxidation. In the longer term, it enhances ATP generation by promoting mitochondrial biogenesis (7, 84), as well as promoting the specialized form of autophagy called mitophagy (18), by which dysfunctional mitochondria are engulfed in autophagosomes and transported to lysosomes where they are broken down to recycle their components. Recycling of bulk cytosol and organelles by autophagy is a general response to starvation, which is also activated by the AMPK ortholog in yeast (70). In mammalian cells, an initial trigger for autophagy appears to be the phosphorylation by AMPK of the kinase ULK1 (18, 40), which then phosphorylates and activates VPS34, a class III phosphatidylinositol (PI) kinase that generates PI3-phosphate (PI3P) (60). This recruits proteins containing PI3P-binding domains to the membrane that then mediate subsequent membrane trafficking events. VPS34 occurs in several distinct multiprotein complexes; interestingly, AMPK appears to activate those complexes involved in autophagy by phosphorylating beclin-1, while inhibiting those involved in other membrane trafficking events by phosphorylating VPS34 itself; this switch depends on the presence of a third component, Atg14L, in the former complex (39). Thus AMPK may divert membrane traffic (an energy-requiring process) toward the autophagy pathway and away from other membrane trafficking events that may not be essential in cells experiencing energy stress.

**FIGURE 3.**
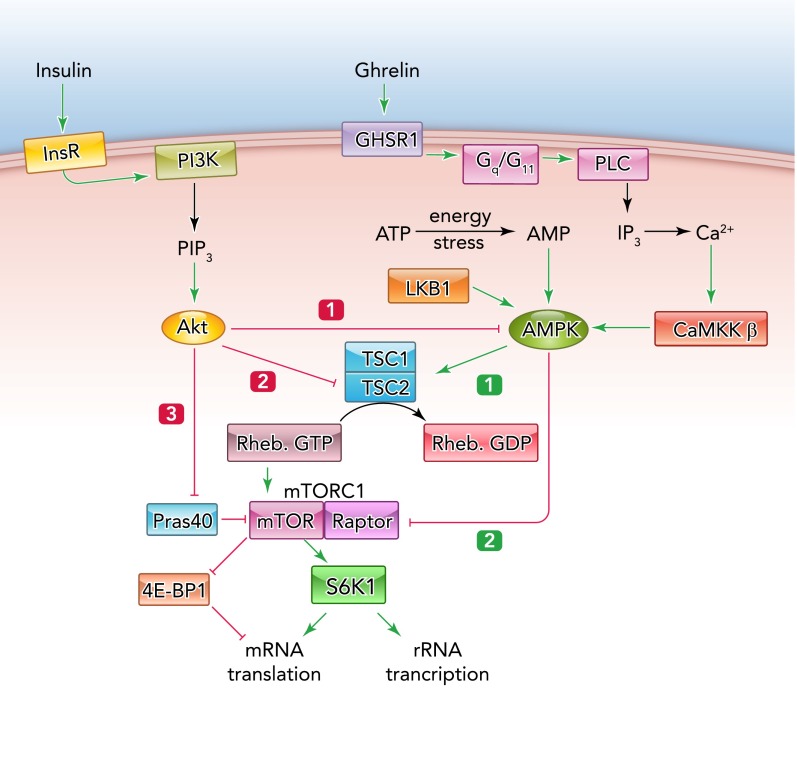
Regulation of mTORC1 by the insulin/Akt and AMPK signaling pathways The insulin receptor activates phosphoinositide 3-kinase (PI3K), causing production of phosphatidylinositol 3,4,5-trisphosphate (PIP_3_), the second messenger that switches on the protein kinase Akt. Akt then has at least three effects: *1*) it phosphorylates AMPK, antagonizing its activation by LKB1; *2*) it phosphorylates TSC2 at sites that oppose its function as a Rheb-GAP, thus activating mTORC1; *3*) it phosphorylates PRAS40, relieving its inhibitory effects on mTORC1. mTORC1 (a complex containing mTOR, Raptor, and other components) then phosphorylates S6K1 and 4E-BP1, promoting cell growth by enhancing translation of specific mRNAs, as well as rRNA synthesis. By contrast, AMPK, which is activated by energy stress or by hormones that increase cytosolic Ca^2+^ such as ghrelin, has two effects: *1*) it phosphorylates TSC2 at distinct sites, enhancing its Rheb-GAP activity; *2*) it phosphorylates Raptor. These effects act to inhibit mTORC1, protein synthesis, and cell growth.

AMPK also switches off almost all biosynthetic pathways, including synthesis of fatty acids, sterols, triglycerides, phospholipids, glycogen, ribosomal RNA, and proteins. These pathways are often downregulated via multiple mechanisms, involving both phosphorylation of key enzymes in the pathway and longer-term effects on gene expression. In the case of protein synthesis, the major action of AMPK is to inhibit the mechanistic target-of-rapamycin complex-1 (mTORC1), a central signaling node that integrates signals arising from growth factors, nutrient availability, and energy status, and promotes cell growth when circumstances are appropriate. mTORC1 promotes cell growth by phosphorylating ribosomal protein S6 kinase-1 (S6K1), by stimulating translation of proteins critical for growth (such as ribosomal proteins) and transcription of ribosomal RNA, and by phosphorylating eIF4E binding protein-1 (4E-BP1), relieving its inhibitory effects on translational initiation (83). Inhibition of mTORC1 by AMPK occurs via dual pathways (FIGURE 3). First, AMPK phosphorylates TSC2 (37), enhancing the Rheb-GTPase activator function of the TSC1-TSC2 complex and thus converting the G protein Rheb, an upstream activator of mTORC1, to its inactive GDP-bound form. Second, AMPK phosphorylates Raptor, a subunit of the mTORC1 complex (22). A similar dual approach is used by the insulin-signaling pathway to activate mTORC1 and thus oppose the effects of AMPK (FIGURE 3). The insulin-activated kinase Akt phosphorylates TSC2 at distinct sites, inhibiting its Rheb-GAP function (36, 47, 59), while also phosphorylating PRAS40, relieving its inhibitory effect on the mTORC1 complex (62). Thus AMPK and Akt exert reciprocal control of mTORC1. Also shown in FIGURE 3 is the inhibitory effect of Akt on AMPK itself, discussed in the previous section.

Recently, O'Malley's group reported that steroid receptor co-activator-2 (SRC-2) was phosphorylated by AMPK and provided evidence that this promoted transcription of mRNA encoding the bile salt export pump, BSEP, in hepatocytes (13). Since transport of bile salts from the liver to the gut is required for efficient adsorption of lipids in food, this represents an interesting mechanism by which AMPK would enhance energy intake into the body.

## Regulation of AMPK by Hormones

Given that it evolved in simple single-celled eukaryotes, the original primary function of AMPK appears to have been to regulate energy homeostasis at a cell-autonomous level. However, during the evolution of multicellular eukaryotes, hormones involved in regulating whole-body energy balance have also developed the ability to regulate AMPK. This final section of the review will focus on the effects of these hormones, whose effects are summarized in Table 1.

**Table 1:** Summary of hormones affects whole-body energy balance and their effects on AMPK

Hormone	Target Cell	Effect on AMPK and Mechanism	Overall Effect
Ghrelin	Neurons in arcuate nucleus	Activation by CaMKKβ	Increased appetite/food intake
Leptin	Neurons in arcuate nucleus	Inhibition (S487/S491 phosphorylation?)	Decreased appetite/food intake
Insulin	Neurons in arcuate nucleus	Inhibition (S487/S491 phosphorylation?)	Decreased appetite/food intake
Insulin	Other tissues	Inhibition (S487/S491 phosphorylation?)	Enhanced biosynthesis, energy storage
Adiponectin	Neurons in arcuate nucleus	Activation via AdipoRI	Increased appetite/food intake
Adiponectin	Skeletal muscle, other tissues	Activation via AdipoRI	Increased fat oxidation/energy expenditure
T3	Neurons in ventromedial hypothalamus	Inhibition by unknown mechanism	Activation of sympathetic nervous system

### Ghrelin

**FIGURE 4.**
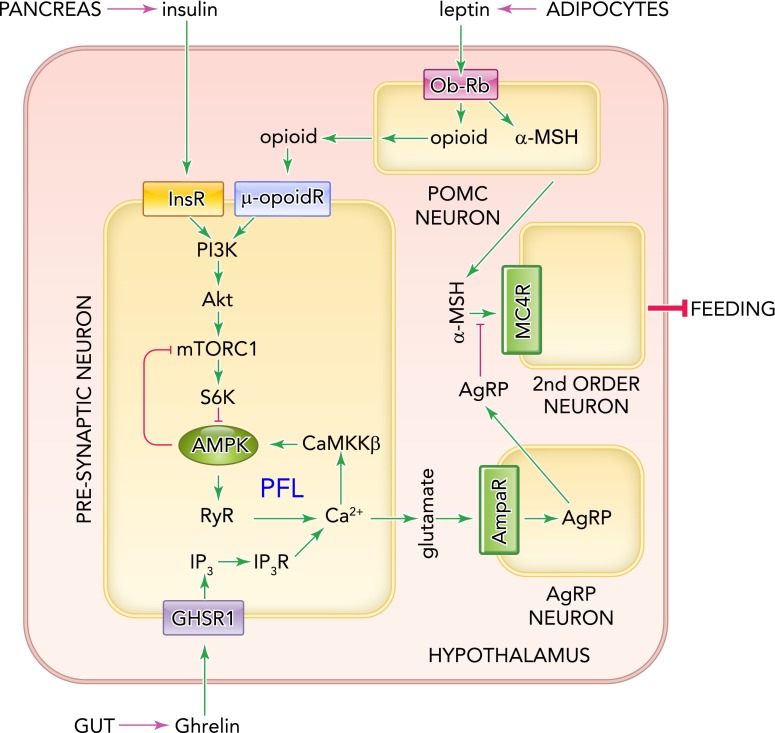
Model for role of AMPK in controlling feeding behavior in mammals, based on Sternson (81) and Kahn (15) According to this combined model, the key role of AMPK is in presynaptic neurons immediately upstream of NPY/AgRP neurons in the hypothalamus. Ghrelin causes release of inositol 3,4,5-triphosphate (IP_3_) in these cells via the G-protein-coupled receptor GHSR1, leading to release of Ca^2+^ via IP_3_, thus activating CaMKKβ and hence AMPK (one effect of which is to inhibit mTORC1 via the mechanisms shown in FIGURE 3). The released Ca^2+^ also triggers release of glutamate that activates the downstream NPY/AgRP neurons, initiating feeding via release of AgRP onto second-order neurons. AMPK is also proposed to activate ryanodine receptors (RyR) that release Ca^2+^, setting up a positive feedback loop (PFL) that allows continuous stimulation of the NPY/AgRP neuron even if stimulation by ghrelin ceases. This loop is interrupted and feeding stops when POMC neurons release an opioid onto the presynaptic neuron, inhibiting AMPK via the PI3K-Akt-mTORC1-S6K1 pathway. Insulin also depresses feeding by activating the same pathway in the presynaptic neuron.

The acylated peptide ghrelin is released from the gut during fasting or starvation and is carried to the hypothalamus, where it acts as a “hunger signal” to increase appetite. Ghrelin activates AMPK in the hypothalamus (2), and there is evidence that this is required for ghrelin to increase food intake (3, 42). Ghrelin receptors co-localize in the arcuate nucleus of the hypothalamus with NPY/AgRP neurons that express neuropeptide Y and agouti-related protein (72), which are required for ghrelin to promote feeding (44). However, recent studies suggest that ghrelin may act not on the NPY/AgRP neurons themselves but on presynaptic neurons immediately upstream (FIGURE 4). The activity of these presynaptic neurons was assessed indirectly in brain slices by measuring the frequency of miniature excitatory postsynaptic currents in the NPY/AgRP neurons (81). Pharmacological analysis of these currents suggested that ghrelin activates the G-protein-coupled receptor GHSR1, triggering production of IP_3_ and consequent release of intracellular Ca^2+^ in the presynaptic neuron. The released Ca^2+^ then triggers phosphorylation of AMPK via CaMKKβ (FIGURE 4). Although the molecular details beyond that point become less certain, AMPK is proposed to activate ryanodine receptors (RyR) that also trigger Ca^2+^ release, setting up a positive feedback loop (AMPK → RyR → Ca^2+^ → CaMKK → AMPK) that allows continued Ca^2+^-dependent release of the neurotransmitter glutamate onto the NPY/AgRP neurons (and hence feeding, via release of α-MSH onto second-order neurons), even after ghrelin release has stopped (81). This model chimes with normal human behavior, since most people continue eating a meal even after the initial hunger pangs have subsided; cessation of feeding instead appears to require the action of a distinct “satiety signal,” with leptin and/or insulin being the prime candidates.

Consistent with this model, increases in food intake induced by administration of ghrelin were absent in mice with a whole-body knockout of CaMKKβ, and the CaMKK inhibitor STO-609 also reduced food intake in wild-type but not CaMKKβ knockout mice (1). However, it should be noted that several other mechanisms linking the hypothalamic ghrelin receptor to AMPK activation and feeding have been proposed (3, 17, 42, 69).

### Leptin and Insulin

The polypeptide leptin is released from adipocytes and can be regarded as a “satiety” signal, indicating that adipose tissue triglyceride stores are replete (19), whereas insulin is released from pancreatic β-cells in response to hyperglycemia. Leptin causes a rapid but transient activation of AMPK-α2 complexes and hence fatty acid oxidation in skeletal muscle, thus increasing energy expenditure (50). Somewhat paradoxically, it has the opposite effect in the arcuate nucleus and other regions of the hypothalamus, where it has been reported to inhibit AMPK-α2 (49). Although leptin is known to bind to Ob-Rb receptors in the hypothalamus, there have been various proposals to explain its inhibitory effects on AMPK. Based on experiments in the brain slice preparation described in the previous section, leptin was proposed to trigger release of an opioid (possibly β-endorphin) from the POMC neurons, which then acts via μ-opioid receptors to inhibit AMPK in the presynaptic neurons upstream of the NPY/AgRP neurons. By interrupting the positive feedback loop that had been set up by ghrelin, leptin would terminate stimulation of the NPY/AgRP neurons and thus feeding (81). If this model is correct, the critical pool of AMPK is in presynaptic neurons (whose exact identity remains uncertain) rather than in the POMC or the NPY/AgRP neurons themselves. This would explain why knocking out AMPK in either of the latter neuronal types failed to abolish the anorexigenic effects of leptin (14).

Another proposal came from findings that intracerebroventricular injection of leptin led not only to decreased AMPK-α2 activity in the hypothalamus but also to increased Ser491 phosphorylation on AMPK-α2. Leptin was also found to activate the PI3-kinase-Akt-mTORC1-S6K1 pathway in the hypothalamus, and S6K1 phosphorylated Ser491 on AMPK-α2 in cell-free assays (15). It was proposed that Ser491 phosphorylation might inhibit Thr172 phosphorylation and consequent activation of AMPK-α2 by upstream kinases. Indeed, experiments involving hypothalamic expression of S491A mutants supported the idea that phosphorylation of this site might be involved in the anorexigenic effects of leptin (15). Since it has been reported that μ-opioid receptors can also activate the PI3-kinase-Akt-mTORC1-S6K1 pathway (57), this model could be combined with the one described in the previous paragraph. An attractive feature of this combined model (FIGURE 4) is that insulin, which is released after a carbohydrate meal and has anorexigenic effects like leptin, also activates the PI3-kinase-Akt-mTORC1-S6K1 pathway and could therefore work via a common mechanism. Indeed, recent studies with GTI-7 cells, a cell line derived from mouse hypothalamus, showed that leptin and insulin both activated S6K1 (71), although in these cells ghrelin had only modest effects on AMPK and did not block the effects of leptin or insulin on S6K1 phosphorylation (63).

Although quite compelling, the model shown in FIGURE 4 is almost certainly over-simplified and is unlikely to be correct in every detail. One caveat is that it has not yet been shown that phosphorylation of Ser491 inhibits the phosphorylation of Thr172, as does phosphorylation of Ser485 on α1 (33). It is also not yet clear whether phosphorylation of Ser485 or Ser491 inhibits Thr172 phosphorylation by CaMKKβ.

We can thus envisage two physiological scenarios in which leptin, insulin, and ghrelin co-regulate food intake and energy expenditure via the neuronal circuits discussed above. During negative energy balance (fasting or starvation), ghrelin increases and leptin and insulin decrease. This will increase AMPK activity in the presynaptic neurons, causing increased activity of NPY/AgRP neurons and decreased activity of POMC neurons, driving a powerful orexigenic output (FIGURE 4). The converse situation, following attainment of a positive energy balance, is associated with rising leptin and insulin and decreasing ghrelin, reducing AMPK activity and increasing anorexigenic outputs. This circuit appears to be dysfunctional in obese individuals who seem to become both leptin- and insulin-resistant in the hypothalamus, allowing ghrelin (even at reduced levels) to escape the restraining influences of those hormones. This would chronically elevate AMPK activity in the hypothalamus, resulting in inappropriately activated NPY/AgRP neurons and maintaining a prolonged hunger signal despite a positive energy balance.

As well as repressing appetite in leptin-sensitive individuals, inhibition of AMPK by leptin in the hypothalamus also causes activation of sympathetic nerves leading to the periphery. One effect of this is to activate AMPK-α2 and stimulate fatty acid oxidation in skeletal muscle, thus increasing whole body energy expenditure, prolonging the more transient direct effects of leptin on muscle (50). These findings suggest that leptin regulates energy balance not only by inhibiting energy intake, but also by enhancing energy expenditure.

### Adiponectin

Although secreted by adipocytes, adiponectin paradoxically displays high plasma levels in lean individuals and low levels in obese individuals, suggesting that it is released when fat stores are low. Adiponectin binds to two related receptors, AdipoRI and AdipoRII (38), and binding to AdipoRI activates AMPK, thus promoting fat oxidation in liver and muscle, and inhibiting glucose production in the liver (80). Adiponectin also increases appetite by activating AMPK in the hypothalamus, mimicking the effect of ghrelin but opposing the effects of leptin (41). However, the molecular mechanisms by which adiponectin activates AMPK via AdipoRI remain unclear.

### Thyroid Hormones

The thyroid hormone tri-iodothyronine (T3) binds to nuclear receptors that regulate transcription and has effects on almost all cells. However, a major effect of T3 on whole-body energy balance appears to involve inhibition of AMPK in neurons of the ventromedial hypothalamus (43). Like the inhibitory effect of leptin, this promotes firing of sympathetic nerves that trigger release of norepinephrine and/or epinephrine in the periphery. This increases energy expenditure by stimulating release from white adipose tissue of fatty acids, which are oxidized in other tissues, and by promoting fat oxidation and heat production in brown adipose tissue (43).

## Conclusions and Perspectives

It is seems likely that the appearance of the AMPK system was a very early event in evolution of eukaryotes, providing a mechanism for the host cell to interact with and cooperate with its new intracellular partner, the mitochondrion. As more complex multicellular eukaryotes developed, this ancestral role as a cell-autonomous energy sensor was retained and even appears to have become more sensitive. Thus the mammalian system is regulated by AMPK by a very sensitive tripartite mechanism, whereas its budding yeast ortholog lacks at least one of these mechanisms (allosteric activation) and also appears to be regulated by ADP rather than AMP. In addition, new systems of regulation have been added on top of these ancestral mechanisms, which allowed AMPK to play new roles in regulating energy balance at the whole body level. Although these additional mechanisms are still being elucidated, they include the ability of the starvation hormone ghrelin to activate AMPK by the Ca^2+^-CaMKK pathway and the ability of insulin, and perhaps leptin, to inhibit AMPK activation by promoting phosphorylation of the ST loop.
